# Man’s best friend in life and death: scientific perspectives and challenges of dog brain banking

**DOI:** 10.1007/s11357-021-00373-7

**Published:** 2021-05-10

**Authors:** Sára Sándor, Kálmán Czeibert, Attila Salamon, Enikő Kubinyi

**Affiliations:** 1 Department of Ethology, ELTE Eötvös Loránd University, Budapest, Hungary; 2 MTA-ELTE Comparative Ethology Research Group, Budapest, Hungary

**Keywords:** Dog, Biobank, Translational research, Big data approach

## Abstract

Biobanking refers to the systematic collection, storage, and distribution of pre- or post-mortem biological samples derived from volunteer donors. The demand for high-quality human specimens is clearly demonstrated by the number of newly emerging biobanking facilities and large international collaborative networks. Several animal species are relevant today in medical research; therefore, similar initiatives in comparative physiology could be fruitful. Dogs, in particular, are gaining increasing attention in translational research on complex phenomena, like aging, cancer, and neurodegenerative diseases. Therefore, biobanks gathering and storing dog biological materials together with related data could play a vital role in translational and veterinary research projects. To achieve these aims, a canine biobank should meet the same standards in sample quality and data management as human biobanks and should rely on well-designed collaborative networks between different professionals and dog owners. While efforts to create dog biobanks could face similar financial and technical challenges as their human counterparts, they can widen the spectrum of successful collaborative initiatives towards a better picture of dogs’ physiology, disease, evolution, and translational potential. In this review, we provide an overview about the current state of dog biobanking and introduce the “Canine Brain and Tissue Bank” (CBTB)—a new, large-scale collaborative endeavor in the field.

## Introduction

Currently, one of the major obstacles to better understand human pathologies and biological processes is the limited accessibility of research material that allows researchers to simultaneously explore different aspects of the same phenomenon. To overcome this limitation in human medical research, biobanks have been established to store pre- and post-mortem biological specimens and offer these samples for research purposes [1–3]. Most biobanks were later organized into international collaborative networks, which was a huge step towards standardized sample collection, quality control, and sample integration [2–6]. These initiatives have advanced fields like neurobiology and aging research [3, 4, 6]. Especially brain research could benefit from specialized biobanks that collect human brain samples. Systematic brain banking has been known since the 1960s [7] and brain banks across the world have since been organized into collaborative groups, like the BrainNet Europe [2, 8]. Samples provided by these organizations have already helped researchers better understand the pathology and characteristic molecular changes of, for example, Alzheimer’s disease (AD), Parkinson’s disease (PD), and amyotrophic lateral sclerosis (ALS). The increasing prevalence of these disorders in modern societies underlines the importance of human brain banking [9, 10].

However, as typical symptoms of most neurodegenerative diseases manifest later than the actual onset of the disease on the cellular level [11, 12], it is challenging to systematically investigate the initial steps of neurodegenerative processes in humans. Most asymptomatic donors may be misidentified as being healthy due to the lack of efficient screening for early indicators of disease development [11–13]. The application of various biomarkers (genetic, neuroimaging, clinical, and biochemical), which could efficiently predict diseases, would be a solution to this issue. However, their validation also requires precise comparisons between the examined tissues and the associated changes in the candidate biomarker [14]. Therefore, the availability of appropriate tissue specimens is a prerequisite for identifying biomarkers of neurodegenerative diseases [15–18].

Importantly, even with suitable markers, human studies still face some major limitations. For example, conducting longitudinal studies to screen changes in markers throughout a human lifespan can be challenging. Both the aging process and the development of neurological pathologies span many years in humans and therefore are hard to be recorded in a standard study setting. This is one of the main reasons why scientists turned to short-lived model organisms to study aging. However, the lack of naturally occurring age-related neurodegeneration and the fundamental differences in living environment and lifestyle between humans and laboratory animals greatly reduces the translatability of findings. Several authors have suggested that these two major limitations of animal models could be addressed by involving companion dogs in dementia research [19–21].

Because of dogs’ unique evolutionary history, living in the close proximity of humans for 15–30 thousand years, convergent evolution is suspected to affect several of their traits. Genes related to environmental adaptation [22] and diet [23] were shown to exhibit similar changes in dogs and humans exposed to the same selective pressures. Other genes showing similar changes can be linked to neurological processes [24, 25]. Finally, there are social abilities dogs possess almost uniquely among animals, which cannot be explained by socialization (to humans) alone [26–29]. As a model species, dogs do not derive value from similarities to humans, alone. Due to artificial selection, dogs have also become one of the phenotypically most variable mammalian species, with a unique population structure and high functional versatility [30, 31]. Through selective breeding strategies and increased inbreeding, a range of heritable disorders [32, 33] has manifested in dog breeds. Many of these show strong similarities to heritable disorders seen in humans making dogs promising translational models. In addition, most age-related pathologies affecting humans have their canine counterparts, including cardiovascular disease, diabetes, obesity, several types of cancer, and neurological pathologies [34–40]. In connection with the breed-specific occurrence of various disorders, the expected lifespan can be dramatically different between breeds. However, it is also correlated with body size in general, even in mixed breed dogs [41].

Dogs are also at risk for developing canine cognitive dysfunction (CCD), which exhibits many similarities with human AD, both in the range of symptoms [42, 43] and in the known neural pathology [44–46]. Companion dogs can therefore be valuable models to study anti-aging interventions and possibly, treatments for dementia [47–52]. However, the accessibility of tissues for detailed pathological and molecular investigations is highly limited in the case of companion dogs. Invasive sampling methods, which may cause the animal pain, suffering, distress, or lasting harm, are morally ineligible in the case of companion animals and are forbidden by current legislation. Even after the animal has died, tissue samples are rarely if ever obtained by researchers, as no infrastructure or nationwide networks exist to serve this purpose. Therefore, obtaining samples has been a major limitation to realize companion dogs’ full potential in research. Especially dementia research would require special sampling methodologies to gain insight into the actual pathological processes ongoing in the brains of CCD-affected dogs. Defining methodological pipelines to collect, store, and distribute brain samples from dogs, offering a reliable basis for researchers, therefore, should be a major goal to allow the utilization of the canine model in this field.

## State of the art in dog biobanking

Dog biobanking, following human examples, could help overcome the obstacles to in-depth pathological and genomic research in pet dogs. Biobanking of domesticated animals has already been suggested as a field with great scientific opportunities [53]. Efforts to establish networks of veterinary biobanks have been reported around the globe [54]. Most of these institutes focus on sample collection and research related to farm animals to support breeding strategies for increased productivity or to help fight disease outbreaks [54]. However, examples like the establishment of a pig biobank dedicated to diabetes research [55] indicate that there is a need for institutes, which solely focus on providing animal samples for translational purposes.

Therefore, the especially high potentials of domestic dogs as translational models may also advance the development of dog biobanking approaches. Traditionally, scientific research mainly relied on laboratory dogs specifically bred and kept for such purposes. Most of these dogs belong to the beagle breed and often come from highly inbred populations [56]. Laboratory dogs are important translational models in pharmacology, and in spite of continuous developments in replacing them and reducing their numbers in accordance with the 3R [57] principle, they are still indispensable. So far, several studies have relied on laboratory dogs to investigate molecular level changes related to aging and disease [42, 58–60]. Therefore, it may seem rational to base tissue sample acquisition on laboratory dogs as their euthanasia can be planned far ahead and allow swift post-mortem sampling. However, this approach holds many limitations. First, the variability of *Canis familiaris* as a species is not represented by laboratory beagles. They do not cover the full genetic, phenotypic, and even behavioral spectrum of dogs and hence do not allow scientists to investigate specific questions related to this variability [61]. For example, a wide variation in expected lifespan between breeds or, in general, dogs with different body sizes could represent a valuable opportunity to search for the genetic and physiological determinants behind this phenomenon. Also, the manifold diseases and phenotypic characteristics of various breeds cannot be found in typical laboratory dogs. For example, several diseases occur only in specific breeds, and these pathologies often show high similarities with rare human disorders, offering promising translational research potential [62]. Second, laboratory dogs live in a restricted environment in terms of social contact and diet. In contrast, companion dogs live together with humans, many residing within human homes, which means their diet, exercise, stressors, and many other biologically relevant factors can mirror the natural range of circumstances, which humans are exposed to [63]. In connection with this, companion and laboratory dogs show marked differences in behavior [61], and this can affect the assessment of cognition, e.g., in canine dementia research. Furthermore, pets, including dogs, have obtained a highlighted role as human companions in modern societies leading to more pronounced emotional bonding reported by their owners. For example, in a dataset collected for investigating empathy, 40–60% of dog owners, even parents, claimed that their dog is more important to them than any human [64]. In a representative poll of a marketing research company, 34% of parents living in the USA said that their pet was their favorite child [65]. Consequently, these owners are often highly motivated to finance expensive care and diagnostic processes to maintain the health of their pets. This has led to the quick development of veterinary care systems and diagnostic pathology. Veterinary databases have been established to provide a reliable and uniform system to link together data belonging to the same animals [66–69], opening the possibility to implement similar big data approaches as known in human medical research [70]. In addition, genetic testing services are commonly used by pet dog owners to assess the risk of various genetic disorders, to determine the genetic relatedness of their dogs, and to support breeding strategies in the case of purebred dogs. The tremendous amount of data gained through these commercial testing services can also hold great potentials for research purposes if it can be linked with phenotypic data belonging to the tested dogs through genome wide association studies (GWAS) [71]. A vast number of GWAS studies have already proven the potential of dogs in discovering the genetic background of traits and diseases [72–74]. However, GWAS studies mainly rely on DNA isolated from non-invasively collected buccal swabs from living dogs, while the variance in gene expression patterns and regulatory mechanisms remain unrevealed.

Based on these aspects, biobanks focusing on companion dogs may hold greater scientific potentials for exploring correlations between different levels of genetic, phenotypic, and environmental variables than traditional research relying on laboratory dogs. So far, a few examples have been reported as biobanking services, which acquire biological specimens from companion dogs. Currently, tumor banks represent the front line in scientific purpose canine biobanking. Many tumor types seen in dogs show strong similarities with their human analogs regarding the predisposing genetic factors and the characteristic pathological features [34, 75]. For example, the efficacy of novel treatments can be assessed in dog patients affected by the tumors analogous to human cancer targeted by the actual drug(s) [40, 76, 77]. The systematic tissue collection and analysis provided by canine tumor banks can facilitate these approaches as they allow insight into actual, cellular level pathological processes and the efficacy of therapies. This way, these initiatives can benefit both veterinary and human medical research. However, tumor banks cannot support the full range of biological research in dogs, as they usually focus on obtaining cancerous tissues and perhaps some of their healthy counterparts. Therefore, studies investigating other pathologies may barely benefit from tumor banks.

Other examples of dog biobanks are organizations, which provide specific tissues for transplantation purposes in the veterinary practice [78, 79]. Importantly, these often rely on post-mortem samples obtained from voluntary donations, made by dog owners whose dogs had to be euthanized for medical reasons. Such voluntary donations are also accepted by several veterinary universities for educational purposes. These facilities represent a valuable source of research materials as well because the protocols and ethical guidelines for voluntary donations and their management are already established. Therefore, active collaborations between veterinary clinics and biobanks can be expected to further increase access to post-mortem samples for scientific goals.

For example, the first canine epigenomics and transcriptomics database, BarkBase, was based on tissue samples obtained from five donated pet dogs [80] at the Cummings School of Veterinary Medicine, Tufts University, USA. Another research-centered biobank in Finland focuses on the investigation of rare canine diseases [62], supporting the notion that there is an increasing need for in-depth transcriptomics and epigenomic research in dogs, especially when they show specific conditions.

Interestingly, the existence of these biobanks was reported as part of actual research papers [62, 80], while no papers were dedicated to describe the establishment and refinement of each in detail. Contrary, in the case of human biobanking, it is common to publish methodological papers about biobank establishment/development [5, 81–83]. Given the scarcity of canine biobanks, reports about the detailed methodologies, experiences, and validation processes would be crucial to support further advances and international collaborative efforts. Specific needs based on increased research demand, e.g., the involvement of companion dogs in dementia research can facilitate the establishment of biobanking services, which, however, should be based on clear and well-defined protocols.

For example, a well-designed brain banking system, capable of gathering a large sample population of CCD-affected dogs with known medical background and lifestyle parameters, would be crucial to uncover all the relevant risk factors and early indicators of CCD in dogs. Ghi et al. (2009) recognized the need for a systematic, long-term collection of brain tissue and accompanying behavioral data from dogs, which represent different stages of aging and CCD progression, as it was impossible to obtain an appropriate sample size within a standard project time-frame to search for associations between behavioral and molecular factors in aged, CCD-affected individuals [84]. Furthermore, brain samples can support several other research goals.

Characterizing the variance in transcriptomic and proteomic profiles of dog brain regions could be particularly relevant for uncovering the genetic regulatory mechanisms responsible for the behavioral variance of dogs. Many canine behavioral abnormalities show high correspondences with their human counterparts, like aggressive tendencies [85], attention deficit hyperactivity disorder (ADHD) [86], obsessive-compulsive disorder (OCD) [87], and even autism spectrum disorder [88]. Studies have already demonstrated that orthologous genes show similar tissue expression patterns in dogs and humans [89] and some polymorphisms in neuroreceptors were linked to similar behavioral variation in the two species [90]. Moreover, the genetic regulatory networks behind the wide range of genetically determined, breed-specific behaviors [91] could also be more easily revealed by comparative gene expression studies than by GWAS.

In addition, breed-specific diseases could be better understood if different biological levels of organization (cell, tissue, and organ) of the affected area could be investigated simultaneously. Biological specimens provided by biobanks could be indispensable to gain a better insight into the mechanisms that lead to such diseases. Epilepsy, for example, is rather common among dogs (general prevalence is 0.5–5.7 %; see [92]), and certain breeds carry an increased risk of epilepsy with prevalence reaching up to 18% [93], indicating the presence of genetic risk factors. Yet, only a few associated polymorphisms have been described so far [94, 95]. Studies that could directly investigate alterations in the transcriptomes and metabolomes of affected dogs’ brains could be especially useful to pinpoint genetic causes.

An example of a recently established initiative to systematically collect brain—and other tissue—specimens from dogs comes from our own laboratory, with a special focus on canine aging and dementia research [96]. The Canine Brain and Tissue Bank (CBTB) has been established within the frames of the Senior Family Dog Project, to support its research goals and to set the fundaments of a repository of biological specimens, which could be distributed for other research groups on demand. It is located at Eötvös Loránd University, Budapest, Hungary, and utilizes the dissection room previously built for the Anatomy Department of the Biology Institute. All necessary instruments are available at the site or can be used in collaboration with other departments located in the same building. The core staff includes a veterinary anatomist (KC), who performs the dissections, and two biologists (including SS), who participate in the sample management and are responsible to supervise subsequent steps in sample processing (e.g., removal of the RNAlater supernatant before deep freezing). Students or other researchers occasionally partake in the sampling events, providing support with simple tasks, like administration of sample IDs. Logistical and communication management (answering owners’ contact e-mails, making arrangements with veterinary clinics and owners, updating databases, etc.) is a shared responsibility of EK, KC, and SS, depending on availability and schedule. Donations are accepted in every case when the necessary legislation criteria are fulfilled, and cases are subsequently categorized based on post-mortem interval, medical history, and other factors. Up to the date of manuscript submission, the Canine Brain and Tissue Bank (CBTB) has collected brains and other tissues (e.g., skin from the head and temporal muscle) from 130 donated cadavers and actively supported current ongoing research projects, including international collaborations. Since its establishment, the CBTB has gone through several steps of protocol development and testing, especially regarding the recruitment of donations and data management. Further developments are being actively considered, especially based on the experiences gained since establishment. For the first, explorative years of the CBTB, the low rate of incoming donations allowed the staff to put more focus on protocol developments and on other, downstream research projects. So far, correlation between *CKDN2A* gene expression and age and ß-amyloid levels and age/cognitive function have been reported based on samples collected by the CBTB [97, 98]. In the following, we will present the latest protocols and experiences of the CBTB.

## Canine Brain and Tissue Bank: perspectives and challenges

To fulfill its supportive role in canine genomics, a dog biobank should systematically collect and store biological specimens, together with related (ideally life-long) medical data, from a wide range of euthanized pet dogs. The main focus of the CBTB has been to collect and store brain-derived samples (ranging from full hemispheres to specific areas) in a way that ensures reliable quality and reproducibility. Beyond this goal, however, the donation events offered possibilities to obtain samples from any other body part, depending on research interest. In this regard, clearly defined priorities should be set to ensure the optimization of the sample collection protocol.

### Sample acquisition and quality

The quality of obtained tissue specimens is a major concern, especially in the case of brain banking. Neural tissue is particularly sensitive to environmental changes and starts to rapidly decompose after death [99, 100]. Several factors affect the quality of brain samples, such as post-mortem interval, pH value, and the grading of autolytic degradation of the granule cell layer [101, 102]. The acceptable range for these values depends in part on the research goals for which they are collected. DNA, for example, is more stable than RNA or most proteins [2]. As the CBTB has been established to provide samples for a wide range of investigations related to the brain, including gene expression studies, the interval between the death of each animal and sample fixation was limited. Based on the human literature [103, 104] and practical considerations (towards conducting molecular research), the post-mortem interval for collecting brain tissue samples was set to 4 h in the CBTB. The developed donation system is allowed to maintain a narrow interval between the euthanasia of the animals and the fixation of samples in most cases. As the CBTB staff are in contact with most of the owners prior to the day of euthanasia, the timing of the donations can usually be planned in advance. When an animal gets euthanized in the prearranged time, a member of the CBTB with valid transport certificate arrives at the clinic and transports the cadaver to the facility of the CBTB. The dissection starts immediately after the arrival of the cadaver (usually 1–1.5 h post-mortem), meaning that on average, the brain tissue can be fixed within 2.5–3 h after the death of an animal.

Donations, for which the sampling time exceeded this limit, were considered for histological and anatomical research (e.g., brain cell counting) purposes only, and the brain tissues were fixed by phosphate-buffered saline-formaldehyde solution. Therefore, currently, two main forms of sampling protocol are performed by the CBTB, based on the elapsed time from the death of the animal. Protocol variant A (Table 1) was performed in donations with < 4 h post-mortem interval. Every other case underwent protocol B (Table 1). According to protocol A, brain regions and parts were distributed to gain both molecular grade and histological purpose samples from the same animals. First, the brains were halved in the midsagittal line. Tissue pieces from pre-defined areas of the brain, similarly to the “standard brain block” defined by Vonsattel et al. [81], were obtained. However, contrary to Vonsattel et al., CBTB samples were isolated from both hemispheres to allow the collection of corresponding probes from the same regions of each animal for methodological testing. To allow optimal penetration of the solution, an analytical scale was used to obtain 80–120 mg of each brain block to be immersed in 1 ml RNAlater, according to the manufacturer’s (Thermo Fisher Scientific) protocol. Although snap freezing is a common method used in human biobanking, RNAlater was chosen as a more convenient method for the stabilization of the relatively few standard tissue blocks obtained within the current, initial state of the CBTB protocol. Importantly, the RNAlater reagent was generally reported to provide reliable preservation of RNA content in tissues and cells [105, 106]. Also, RNAlater was shown to provide superior protection for samples in cases of freeze-thaw cycles [107]. However, for the reliable preservation of larger/more numerous samples, snap freezing should be considered as the less expensive and more common method, which could be applied based on the examples of human brain banks.

**Table 1:** Main elements of the two protocol variants currently applied in the Canine Brain and Tissue Bank

Post-mortem delay	Protocol	Brain parameters (dimensions and weight)	Brain halving	Hemisphere fixed in 4% buffered formaldehyde solution for histology	Standard brain samples in RNAlater for molecular purposes	Hemisphere sliced and blocks fast frozen (−80 °C) for molecular purposes
< 4 hours	A	Yes	Yes	One	Yes	Yes
> 4 hours	B	Yes	Yes	Both	No	No

Another methodological challenge, which should be addressed in canine brain banking, is the optimal slicing of the fresh brain to allow access to inner regions and nuclei. Slicing the fresh brain with reproducible cutting distances is challenging without proper instruments, also in the case of the human brain. For example, Vonsattel et al. described a slicing method with a specifically designed instrument, which allowed to produce 0.5-cm-thick coronal slices of a human cerebral hemisphere [81]. Because dog brains are much smaller than human brains (the average dog brain volume is approximately 6% of the average human adult brain, see, e.g., [108]), the optimal thickness of obtained slices should be reduced if regions are to be accessed with the same resolution. So far, due to the still preliminary nature of the CBTB as a canine brain bank, slicing methods have not yet been developed to allow the acquisition of fresh brain slices with the resolution reported in human brain banking. In the currently applied protocol, the hemisphere intended for molecular purposes is divided into larger parts following anatomical borders (e.g., separating regions in the lines of common sulci). These parts are then frozen separately for later usage.

Another factor, which could be specifically relevant in canine biobanks, is the fact that most donated dogs are euthanized by chemical injections. It is known that chemicals (most commonly T-61) used to euthanize animals result in severe hemolysis [109] and rapid changes in brain physiology before death [110]. For this reason, blood samples from euthanized dogs were not considered for sensitive molecular research purposes in the CBTB and rather the pre-mortem collection of blood was inquired from the veterinarians when it was possible. However, it is also unclear how such changes would affect molecular components in the brain. Therefore, dog biobanks should endeavor to evaluate the occurrence of possible systematic artefacts resulting from euthanasia. For this purpose, sample pairs from easily accessible tissues and body fluids (e.g., blood) taken from the same animals before and after death would be highly valuable.

### Collaboration with veterinarians and owners

**Fig. 1:**
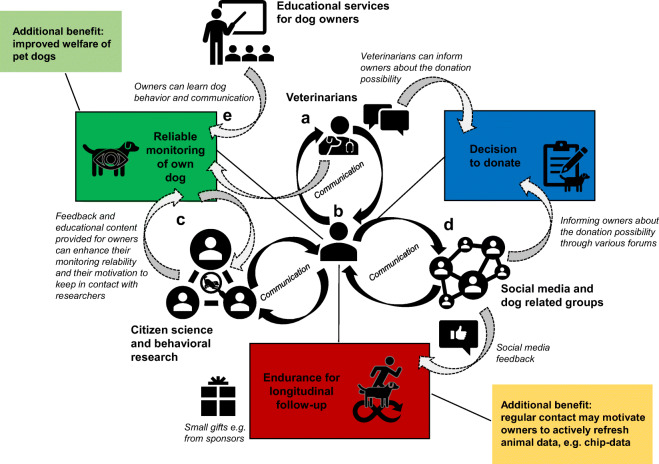
Involvement of dog owners in dog bio- and databanking**. a** Veterinarians are in regular contact with owners; therefore, they can inform them about participation possibilities in research, if they themselves are involved in related networks. Veterinarians are also the main providers of reliable medical information about the animals. **b** Dog owners make decisions whether to participate in behavioral experiments and/or donate the cadaver of their animals after euthanasia for medical reasons. Therefore, effective communication strategies should target them first. Also, a well-organized and General Data Protection Regulation (GDPR) compliant interdisciplinary network of researchers from different fields, focusing on the owners as caretakers of animals, can reduce the effort required from owners by diminishing the redundancy of collected data. **c** Citizen science and behavioral research both requires effort from owners, either by collecting data (e.g., experiments) at home or by traveling to research facilities with their dogs. Educational and social content, together with specific freebies (dog food, toys), provided by the researchers can compensate this effort. **d** Social media can open up several methods of effective communication with groups of owners and allow researchers to reach a wider audience than by informing owners directly during tests/veterinary visits. In addition, social media can also provide networking interfaces to connect professionals from various fields. **e** Owners can provide valuable information about the lifestyle and behavior of their pet dogs, and the more educated they are in recognizing dog behavioral variants and communication signals, the more reliable their provided data can be

Interdisciplinary collaborative approaches, together with well-defined sampling procedures and data management guidelines, have been shown to facilitate longitudinal data integration and sample collection in the case of human biobanks [111–116]. Biobanking could be involved in all steps of clinical research and healthcare, both in human and veterinary medicine, and can contribute to more patient-centered healthcare and reduce the overall cost of clinical trials [5]. Therefore, active collaboration and communication with veterinarians is not only a major prerequisite but could also benefit both parties (Fig. 1a). As a first step, veterinarians directly contribute to tissue banking through the donation process. The veterinarians are responsible for euthanizing the dogs according to the general clinical guidelines for humanely euthanizing companion animals. In the case of the CBTB, the euthanasia is exclusively performed by collaborating veterinarians at veterinary clinics, which are located within a maximum of 1–1.5-h drive from the CBTB facility. Since the host institute does not include veterinary education and a related teaching clinic, permissions for veterinary care services and pet animal euthanasia are not available. As a second reason, in our experience, most owners choose to euthanize their pet dogs at a familiar clinic or at home and by a veterinarian whom they know and trust. Consequently, the protocol of the euthanasia may vary depending on the veterinarian who performs it according to individual professional experience and decision. However, all data about the chemical agents used for sedation and euthanasia are recorded on the consent form; thus, these factors can be controlled during subsequent analyses, if necessary. Apart from this inconsistency in the applied procedures and drugs, collaboration with third party veterinarians can have several advantages. Veterinarians performing the euthanasia have to officially state in the donation consent form that the euthanasia was justified by medical reasons and the dog did not suffer from medical conditions, which would be hazardous to the personnel performing the sample collection (e.g., rabies). Therefore, a veterinarian who had been familiar with the donated dog can provide a more detailed insight into the medical history of the donated dogs. Third-party veterinarians could also inform dog owners about the donation possibility (Fig. 1b), increasing the number of potentially informed owners. In turn, veterinarians—or owners—can receive feedback about the pathological state of the animal, which could be assessed during the dissection performed by the CBTB personnel, and samples can be sent for more detailed pathological examination on demand from the veterinarian or the owner.

**Fig. 2:**
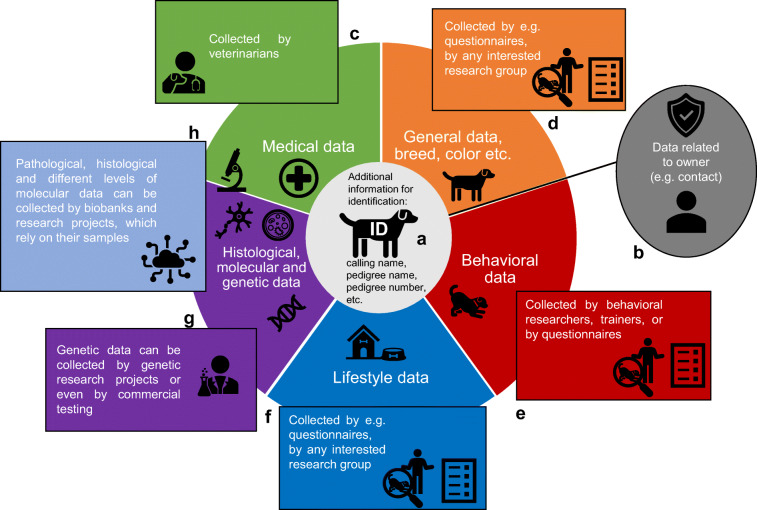
Data types, methods of data collection, and research groups that could be linked to animal biobank data. **a** The scientific applicability of samples stored in a biobank mainly depends on the range of data linked to the specimens. While some information can be gathered about the animals following donation, medical data, lifestyle data, and other levels of related information should be collected during the life of the animal or by asking the owner following donation. Therefore, a reliable identification system with consequent individual IDs is crucial to link all related data to the animals, especially if it is gathered longitudinally through the lifespan (e.g., medical data). **b** Medical data about the donated animals could be gathered from veterinarians or veterinary databases with permission. Also, some medical questions can be covered by owner reports; however, reliability should be taken into account if such data is used in an analysis with a small sample number. **c** Phenotypical, breed- and line-related data could be collected by asking the owners or by accessing pedigree databases in the case of pedigree dogs. Pedigree name and other related IDs can also serve as secondary identifiers of animals. **d** All information linked to the animals’ owners should be stored separately from data related to the animals. Data integration in this case can only be provided if dogs are identified by a reliable and consistent ID, which can be accessed by any investigator at any time in the same way. **e** Behavior is a special form of phenotypic data, as it usually requires professionals or individuals trained to efficiently recognize dogs’ communication signals for standardized characterization. Owners may be involved in behavioral assessment through citizen science networks, or dogs can be directly tested by professionals. **f** Lifestyle data, including living environment, diet, and training history, can be collected by asking owners, as reliability issues may be less concerning in this case. This type of data could be highly valuable to exploit the potential of companion animals in translational research. **g** Genetic and other molecular data can either be gathered from pre- or post-mortem sampling. As more and more dogs’ samples are investigated by commercial genetic testing services, involving these results in research can eventually reduce data redundancy and can be more cost-effective for research projects. This requires well-established collaborative networks with good GDPR and data-sharing policies. **h** Post-mortem samples derived by biobanks can be the main source for detailed multi-level molecular analyses. Also, histological and pathological information may be directly provided by veterinarians, who treated the animals prior to euthanasia

The veterinarians who know and treat the dogs throughout their lifespan can also partake in longitudinal screening projects of live animals, which, probably, will be donated at the end of their lifespan. Interdisciplinary collaboration and involvement of veterinarians in longitudinal screening efforts represent the most effective way to gather pre-mortem samples (e.g., blood or some tissues if the animals have to undergo veterinary examination or specific surgeries) and different levels of data from companion dogs (Fig. 2), which may develop various pathologies during the screening period (ideally the whole lifespan). Veterinary databases could represent a highly valuable source of medical data as most veterinary clinics utilize practice management software [117].

On the other hand, data about behavior, lifestyle, diet, daily routines, and training, which would be especially relevant in CCD research, would not be typically available in this way. This limitation could be overcome by involving dog owners in the data acquisition (Fig. 1b). Owners may be able to reliably characterize their dogs’ basic behavioral parameters by filling in questionnaires, which had been previously validated to effectively describe the actual behavioral characteristics of dogs [118]. Also, owners can continuously monitor their dogs throughout their lifespan (Fig. 1c) allowing researchers to gather otherwise inaccessible data about lifestyle and daily routines. Importantly, dog owners also have to make the hard decision to donate the body of their pet animal. Therefore, they represent a key contributor to both data acquisition and study design altogether (Fig. 1b).

Therefore, addressing the owners in a suitable way represents a key question for both big data approaches and biobanking itself. If owners, who partake in research projects (e.g., behavioral tests) or take their dogs to the veterinarian, are properly informed by professionals about the significance of canine research, longitudinal follow-up, and voluntary donations, they may decide to contribute to such projects and even donate their dogs’ bodies when the time comes. In this regard, experiences gained from human biobanks on how to address possible donors may be useful to develop communication strategies towards dog owners. In general, both public awareness and patient-centered communication were reported as main factors that determine willingness to donate in the case of human biobanks [119]. Lin et al. (2019) identified four factors that may determine a person’s brain donation decision [120]: contextual knowledge, conceptual understanding, family/friends’ opinions matter, and personal experience, time, and process. However, these factors interconnect to inform the complex psychological and social processes behind a person’s decision. Similarly, complex factors may determine the willingness of dog owners to donate their pets’ bodies; however, this is a question yet to be investigated, as the human-dog bond seems to possess several unique features. For example, owners tend to attribute more emotions to their dogs than to their cats in general [121] and they tend to open up emotionally more easily towards their dogs than to other people [122], indicating that pet dogs could play a very important role in their social and emotional life. Therefore, it is plausible that somewhat different considerations underlie the decision of donating a pet dog’s body than donating one’s own or a family member’s body. Altogether, involving more owners in dog biobanking may require specific communication forms. So far, behavioral research has been the main field which relied on the voluntary participation of dog owners, and the parent institute of the CBTB has vast experience utilizing social media platforms to recruit owners. While social media-based advertisement of biobank donation may seem somewhat extraordinary, it is a viable option in the current times [123], and this approach has already brought success in recruiting human donors [119]. Many social media groups exist with dog-related topics, and these can open an effective way to inform owners about research and donation possibilities (Fig. 1d).

Social media platforms can also augment the active involvement of dog owners in data collection throughout the lifespan of their dog (Fig. 1c). The manifold benefits of involving owners in the longitudinal screening of dogs have already led to the initiation of citizen science approaches to gather behavioral data from a large number of pet dogs [124–127]. Well-designed citizen science methods can also provide feedback to the owners and invite them to actively discuss issues with their dogs in specific social forums, where professionals are also present. By motivating owners to more actively monitor their dogs, citizen science can indirectly support other goals, which would require regular administrative activity from owners, like routinely checking chip number associated data and providing necessary updates [128]. However, introducing citizen science to acquire behavior and lifestyle data in connection with biobanking may hold several challenges. First, most owners are not trained professionals and may assess their dogs’ behavioral parameters differently than a researcher/trainer/handler would do. Also, owners may also fail to consistently reproduce the experimental settings at their homes, resulting in less reliable data than in the case of a conventional method, i.e., behavior tests performed by professionals. In large-scale behavioral studies, where data is obtained from hundreds of pet dogs, the noise caused by such suboptimal settings could be reduced by statistical methods. However, if owner-reported data is collected for a limited number of donated animals, the detection accuracy of citizen science approaches should be considered an important factor. For example, in the case of CCD, a large proportion of cases are not diagnosed in the early stages of dementia because owners do not consider minor behavioral changes important [129]. In this regard, educating owners about dog behavior and communication signals, in general, could both benefit the welfare of companion animals and enhance the reliability of owner-reported behavioral assessments (Fig. 2c). Therefore, collaborative and longitudinal screening approaches linked to canine bio- and databanking should also involve initiatives to support public education (Fig. 1e) in related topics (e.g., canine healthcare, owner’s responsibilities). Human examples have shown that providing educational content to patients (e.g., online games) [130] positively affected their willingness to sign up as donors. In the case of the CBTB, the parent institute regularly holds free access scientific conferences to provide popular science content for owners who follow the research group and often partake in experiments or have donated their dogs’ bodies. This indicates that long-term contact with owners provided by social media, research, and educational programs could create a reliable fundament for biobanking linked big data approaches.

### Data management

Both the successful involvement of dog owners in longitudinal data acquisition and the active collaboration with veterinarians to obtain pre-mortem biological samples would mean an increasing number of data layers managed by a dog biobank. Therefore, the greatest challenge of big data approaches linked to biobanking is to efficiently integrate data derived from various independent sources. Connecting and managing different levels of data is one of the greatest challenges in human biobanking and related research projects, and this has led to the development of several software tools to support data integration [114, 130, 131]. Similar software tools could be advantageous to support data integration in canine tissue banking too. In the case of the CBTB, donated animals are given an ID number based on the date of donation, and all sample identifiers are linked to this ID. Currently, data derived from independent sources (e.g., medical data) is added manually to the biobank registry. In the future, automatic software tools used to cross-link data in different databases can facilitate these processes. Furthermore, efforts should be made to establish data management systems, which would allow to store and manage all layers of data linked to the same animal through a unique ID already assigned during its lifetime (Fig. 2a). This would allow systematic and more centralized gathering of longitudinal medical, behavioral, and lifestyle data. Such systems already exist in human biobanking [132]. In connection with this, several strategies have been developed for the effective yet anonymous identification of patients [133, 134]. While anonymous identification would not be that important in the case of dogs, data linked to their owners must be treated separately from research (Fig. 2b). As a possible solution, additional layers of data could be integrated into veterinary databases or microchip-registry databases. The microchip number is a tempting solution; however, microchip use in dogs is not mandatory in most countries.

Altogether, integrating various types of information linked to each individual dog (e.g., lifestyle, diet, behavior) could be more challenging compared to humans. Medical data gained from veterinary practices (Fig. 2c) may be relatively easily linked to a biobank entry by connecting the individual IDs used in the two datasets as it has already been done by manually adding entries in the CBTB. Also, basic phenotypic data (Fig. 2d), like birth date, breed, coat color, sex, and neutering status, are usually stored in veterinary databases or could be accessed from pedigree databases in the case of pedigree dogs. However, usually, less focus is put on collecting other, even medically relevant information, like the diet or behavior. As owners may decide to participate in partly or entirely independent research projects during the lifetime of a dog, the linking of all the data gathered is especially challenging. Therefore, most ideally both behavioral (Fig. 1e) and lifestyle (Fig. 1f) data should be specifically collected by researchers, who might be interested in subsequently analyzing these variables together with genetic and molecular variables (Fig. 1g, h) measured in biobank samples. Collecting such information about dogs may be more difficult compared to human patients, who, in most cases, can easily answer medically relevant or lifestyle questions via self-reports even in a retrospective manner. On the other hand, genetic data (Fig. 1g) might be more easily gained from commercial genetic testing results, as these tests are becoming more and more common among pet dogs, to test their ancestry and their genotypes at disease associated or trait determining loci.

In the case of the CBTB, the expertise of the host research team in collecting behavioral data from pet dogs could benefit these efforts. A large dataset of different behavioral parameters is available from dogs participating in different behavioral experiments, and if any of these dogs are offered for the CBTB, the different sources of data can be linked together. Also, behavioral data is gathered in connection with the actual donation events, through questionnaires. Currently, two questionnaires are available for donating owners, who can fill them voluntarily. One is intended to gather basic information about behavior and lifestyle and the other one is used to assess CCD scores (based on the CCDR questionnaire, see Salvin et al. 2011 [135]). Furthermore, the owners can state on the donation consent form, whether they can be contacted by the research team to collect further information about the dogs.

### Distribution of biospecimens and sharing of related data

Due to the relatively small size of the current sample repository, sample distribution is managed manually, and the process is supervised by members of the team (KC, EK, and SS), depending on availability, schedule, and the type of sample request (e.g., molecular specimens are mainly supervised by SS, while formalin-fixed brain samples by KC). No specific distribution protocols, and guidelines have been developed so far; however, this should be a main step in the future developments of the CBTB. Until submission of the manuscript, samples have been used in research projects running within the frames of the Senior Family Dog Project, the parent project of the CBTB, and in a few collaborative projects, including canine dementia research, comparative brain histology, and genomics. Due to the yet limited number of requests, the distribution of samples have not been undergone a prioritization process, and all samples were provided to the first request.

In cases of an actual sample request, the location of each sample is first determined based on the database entries, and then all samples are collected and prepared for transportation. Transportation is planned and managed according to the type of the sample. In the case of frozen tissue pieces, all processing steps are performed on dry ice to prevent accidental thawing and refreezing of the samples and to minimalize temperature changes as much as possible. Accompanying data is shared as extracts (mainly in excel format) from the datasheets used to store our data. The original datasheets are never shared to protect any personal data related to owners.

### Current limitations of the Canine Brain and Tissue Bank

Since its establishment in 2017, the CBTB has acquired brains and other tissue samples from more than a hundred donated pet dogs, which is a relatively small number compared to leading human brain banks [81, 136]. One of the major obstacles faced by the CBTB in its first years was the lack of previous expertise in dog biobanking. For example, the timeframe for the acceptance of donations for molecular purposes was set based on human literature [103, 104], as no detailed information was present on how the time after death would affect the brain and molecular parameters in dogs. Consequently, sampling protocols are continuously under development. For instance, in human brain banking, protocol variants are commonly distinguished based on the pathologies present in the brain (e.g., in [81]), and this could be another step towards refining the canine brain banking protocol. The still ongoing development of sampling protocols may result in a less consequent sample quality since the establishment of the CBTB. This should be taken in account in downstream analyses.

Another major limitation was that some of the instrumentation needed for optimal sample processing was unavailable at the start of the CBTB. For example, a standardized, automatized paraffin embedding scheme is still lacking, which means that the quality of the formalin-fixed samples may be lower than would be expected in a clinically relevant sample collection.

In connection with recruitment, our experience shows that several owners who had been in contact with the parent institute for years and had participated in behavior experiments with their dogs did not decide to donate their dogs’ bodies when the animals passed away. A possible reason could be that the CBTB is still novel, and more time is needed to build trust and efficient information spreading. In this regard, exploring the main psychological factors that affect the decision making of dog owners about donation might be a useful study direction to support both the CBTB and other canine biobanks worldwide.

## Conclusions

Dogs have been proposed as a natural model species with high translational potential to correspond to humans in social and cognitive abilities, aging, disease, and metabolism. There is a need to establish dog tissue banks for veterinary purposes, education, and for the support of translational research; human tissue banks would be good models for this purpose. Dog biobanks could pave the way for canine big data approaches, where the integration of data from multiple fields of science (genetics, physiology, behavior, veterinary science, medicine, etc.) is required to assess the status of the dogs and to decide on further action regarding treatment and the translation of the information/results to humans. Establishing a dog tissue bank has its own challenges from providing good quality research materials and gathering several layers of relevant data to risky financial sustainability, though in return it could benefit the worldwide research community. The full potential of such initiatives could be realized through the longitudinal follow-up of companion dogs to gather a range of non-invasively or even surgically (e.g., during veterinary care, sterilization, etc.) obtainable biospecimens and through the long-term collection of post-mortem donations. Therefore, the establishment of specific financial and supportive funding frameworks may encourage more canine research groups to commit to such long-term and challenging initiatives.
